# Four new Neotropical *Lophocampa* species with a redescription of *Lophocampa atriceps* (Hampson) (Lepidoptera, Erebidae, Arctiinae)

**DOI:** 10.3897/zookeys.264.4166

**Published:** 2013-02-06

**Authors:** Benoit Vincent, Michel Laguerre

**Affiliations:** 1 1 rue Roger RAMEAU 93 110 Rosny sous Bois, France; 2 Correspondant, Muséum National d’Histoire Naturelle, Département Systématique et Evolution, USM 602, case postale n° 50 (Entomologie), F-75231 Paris Cedex 051; 3 C.N.R.S. Institut Européen de Chimie et Biologie IECB – UMR 5248, 2 rue Robert Escarpit 33607 Pessac Cedex, France

**Keywords:** Lepidoptera, Erebidae, Arctiinae, *Lophocampa*, new species, Costa Rica, Venezuela, Colombia, Ecuador, Peru, Bolivia

## Abstract

Four new species of *Lophocampa* Harris, are described and illustrated: *Lophocampa flavodorsata* **sp. n.**, *Lophocampa griseidorsata* **sp. n.**, *Lophocampa herbini* **sp. n.** and *Lophocampa sullivani* **sp. n.** *Lophocampa atriceps* (Hampson) is redescribed, illustrated and compared to the new species.

## Abstract (fr)

Quatre nouvelles espèces de *Lophocampa* Harris sont décrites et illustrées, *Lophocampa flavodorsata* **sp. n.**, *Lophocampa griseidorsata* **sp. n.**, *Lophocampa herbini* **sp. n.** et *Lophocampa sullivani* **sp. n.** *Lophocampa atriceps* Hampson, est redécrite illustrée, et compareé aveć les especies nouvelles.

## Introduction

The genus *Lophocampa* Harris is one of the most speciose of the Neotropical tiger-moths, with 80 species and 10 subspecies described (Vincent 2011). The classification of this complex genus is far from complete and in spite of the work of Watson and Goodger (1986), numerous nomenclatural changes are needed to achieve a better taxonomic understanding of the genus.

After several expeditions to the Andean mountains, we now have good series of the *Lophocampa* species previously treated as *Lophocampa atriceps* (Hampson), described from Colombia. Examination of the male genitalia of specimens from Venezuela to Bolivia shows that several undescribed species are presently included in the concept of *Lophocampa atriceps*. Examination of additional material housed in MNHN showed that five species are involved, four of which were new and described here. *Lophocampa atriceps* is redescribed and compared to closely related species, and a key to the *Lophocampa atriceps* group is provided.

## Methods and materials

Abdomens were removed and genitalia were dissected, examined and mounted on slides using standard procedures prior to being photographed. Nomenclature for abdominal and genital morphology follows Klots (1970). The types of *Lophocampa atriceps* (Hampson), *Lophocampa hyalinipuncta* (Rothschild) and *Lophocampa andensis* Schaus were examined and photographed, the two first ones being not dissected.

Treatment of the Arctiinae as a subfamily of Erebidae follows Zahiri et al. (2012); tribal placement follows Lafontaine and Schmidt (2010).

Collections where material used in this study is located are as follows:

BMNH The Natural History Museum, London, UK

INBio Instituto Nacional de Biodiversidad, San José, Costa Rica

MNHN Muséum National d’Histoire Naturelle, Laboratoire d’Entomologie, Paris, France

MUSM Museo de Historia Natural, Universidad Nacional de San Marco, Lima, Peru

USNM Smithsonian Institution National Museum of Natural History, Washington DC, USA

BSC Personal collection of Bolling Sullivan, Beaufort, North Carolina, USA

BVC Personal collection of Benoit Vincent, Rosny sous Bois, France

MLC Personal collection of Michel Laguerre, Léognan, France

**Molecular Analyses.** Tissue samples of the newly described species were sequenced at the Canadian Centre for DNA Barcoding in Guelph (Ontario, Canada), as part of a DNA barcoding project for neotropical tiger-moths, developed as part of the iBOL Lepidoptera campaign (see www.lepbarcoding.org for details). DNA extraction, PCR amplification and sequencing follow the protocols described in Vaglia et al. (2008).

The taxa included in this study are listed in Table 1. Unfortunately, recently collected material of *Lophocampa andensis* was not available for sequencing. A set of twenty-four specimens of five species was sequenced for the 658 barcode base pairs fragment. Sequences were analyzed using maximum likelihood (ML) and maximum parsimony (MP) methods. MP analyses were carried out with MEGA 5 software (Tamura et al. 2011).

**Table 1.** Distribution of *Lophocampa* species. Max. alt: Maximum altitude; Min. alt.: Minimum altitude; P: Pacific; A: Atlantic.

**Species**	**Country (Province)**	**Min. Alt. (meters)**	**Max alt.(meters)**	**Slope**
*Lophocampa atriceps*	**Colombia** (Valle del Cauca), **Ecuador** (Guayas) and **Costa Rica** (Alajuela, Guanacaste, Puntarenas, Cartago).	700	1450	P
*Lophocampa flavodorsata* sp. n.	**Venezuela** (Aragua, Lara, Merida), **Ecuador** (Napo, Morona-Santiago, Sucumbios), **Peru** (Cuzco, Huanuco) and **Bolivia** (Cochabamba, La Paz)	1000	2200	A
*Lophocampa griseidorsata* sp. n.	**Ecuador** (Napo, Morona-Santiago, Sucumbios, Azuay), **Peru** (San Martin, Cuzco, Pasco), **Bolivia** (Cochabamba, La Paz)	1400	2100	A
*Lophocampa herbini* sp. n.	**Peru** (Cuzco), **Bolivia** (Santa Cruz, Cochabamba, La Paz)	2000	2500	A
*Lophocampa sullivani* sp. n.	**Colombia** (Valle del Cauca), **Ecuador** (Pichincha)	1840	2000	P
*Lophocampa andensis*	**Colombia** (Cundinamarca)	-	-	A
*Lophocampa hyalinipuncta*	**Ecuador** (Morona-Santiago, Carchi), **Peru** (Huanuco, Puno, Amazonas), **Bolivia** (La Paz, Cochabamba, Chuquisaca)	2000	3350	A

## Systematics

### Key to species of the *Lophocampa atriceps* species-group

**Table:** 

1	Dorsal surface of abdomen with long yellowish hair; last spot on subterminal band with ovate apex and compressed base (fig. 25a); Distance between last apical spots of the postmedial and subterminal bands greater than the diameter of the apical spot of the subterminal band (fig. 25b)	2
–	Dorsal surface of the abdomen with long greyish or brownish hair; last spot of subterminal band with apex ovate (fig. 26a); distance between the last apical spot of the postmedial and subterminal bands less than the diameter of the apical spot of the subterminal band (fig. 26b)	4
2	Patagia with uniformly black spot	*Lophocampa atriceps*
–	Patagia with a yellow spot outlined with brown	3
3	Male valvae long and slender with apex slightly bent; eastern slope of the Andes (fig. 27)	*Lophocampa flavodorsata* sp. n.
–	Male valvae wide and relatively short with apex notched, forming two points, one blunt and one acute (fig. 28); western slope of the Andes	*Lophocampa sullivani* sp. n.
4	Spots of subterminal band significantly smaller than spots of postmedial band (fig. 2)	*Lophocampa hyalinipuncta*
–	Spots of subterminal band same size as spots of postmedial band (fig. 5, 6)	5
5	Length of male forewing less than 20 mm; apex of forewing rounded; spots between M2 and CuA1 at least as large as the other spots (fig. 9b in Watson, 1973)	*Lophocampa andensis*
–	Length of male forewing than 20 mm; apex of forewing pointed; spots between M2 and CuA1 smaller than other spots (fig. 5, 6)	6
6	uncus lozenge-shaped with apex truncated (fig. 12); valvae without a prominent costa, with bifid apex with both tips symmetrical, short and acute (fig. 30); aedeagus moderately narrow and rectilinear (fig. 18); female ductus bursae short, square (fig. 24)	*Lophocampa herbini* sp. n.
–	Uncus spatulate (fig. 11); male valvae with a prominent costa ending in an acute apex. Apex of the valvae bifid; one tip short and rounded, other very long and acute (fig. 29); aedeagus narrow and rectilinear (fig. 17); ductus bursae rectangular (fig. 23)	*Lophocampa griseidorsata* sp. n.

### Species accounts

#### Lophocampa atriceps

(Hampson)

http://species-id.net/wiki/Lophocampa_atriceps

**Figures 1–6.**
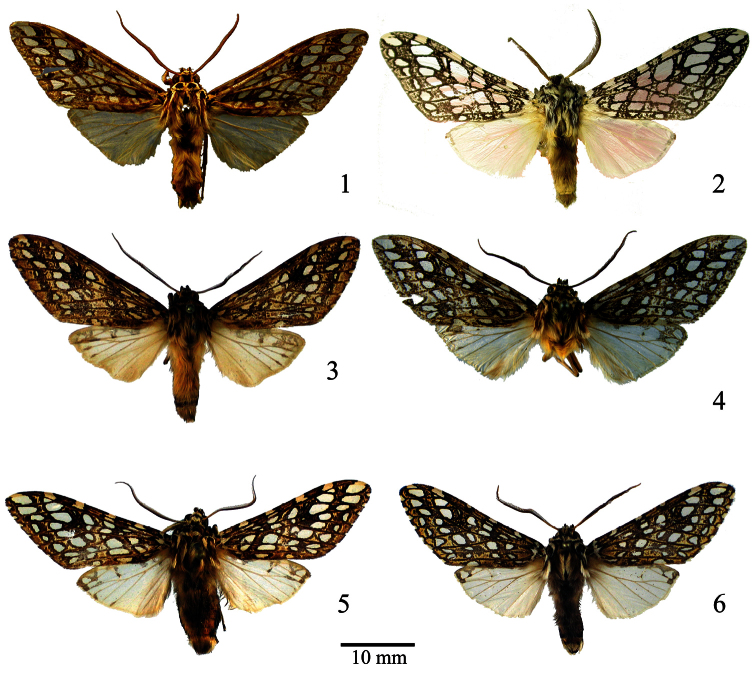
Habitus of *Lophocampa* species. **1***Lophocampa atriceps* (Hampson, 1901), Holotype, male **2** *Lophocampa hyalinipuncta* (Rothschild, 1909), male syntype **3** *Lophocampa flavodorsata* sp. n., Holotype, male **4** *Lophocampa sullivani* sp. n., Holotype, male **5** *Lophocampa griseidorsata* sp. n., Holotype, male **6** *Lophocampa herbini* sp. n., Holotype, male.

**Figures 7–12.**
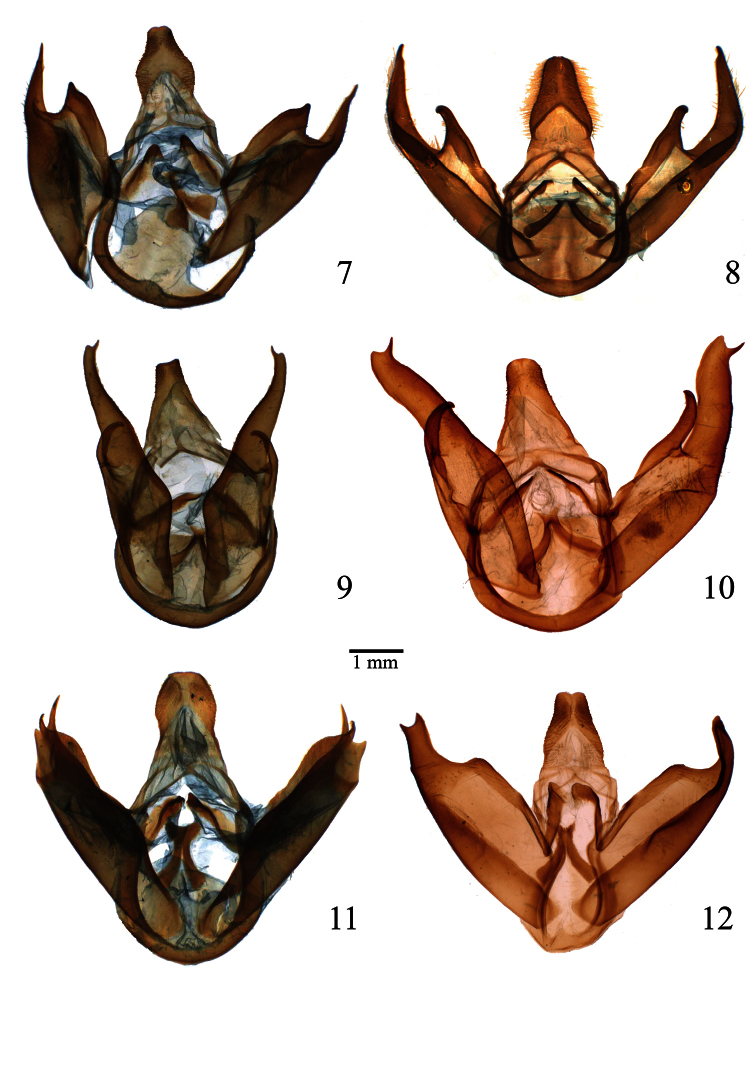
Male genitalia of *Lophocampa* species. **7** *Lophocampa atriceps* (Hampson, 1901), specimen from Ecuador **8** *Lophocampa hyalinipuncta* (Rothschild, 1909), specimen from Ecuador **9** *Lophocampa flavodorsata* sp. n., Holotype **10** *Lophocampa sullivani* sp. n., Holotype **11** *Lophocampa griseidorsata* sp. n., Holotype **12** *Lophocampa herbini* sp. n., Holotype.

**Figures 13–18.**
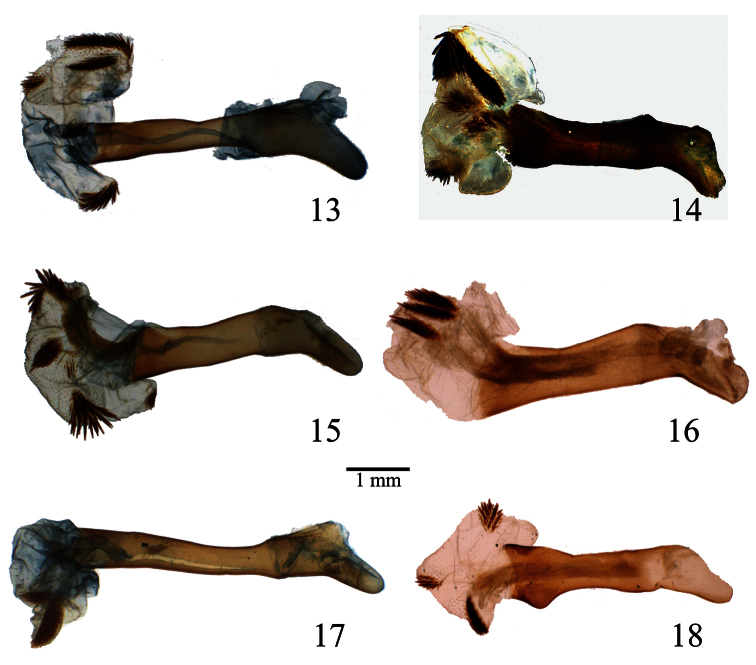
Penis of *Lophocampa* species. **13** *Lophocampa atriceps* (Hampson, 1901), specimen from Ecuador **14** *Lophocampa hyalinipuncta* (Rothschild, 1909), specimen from Ecuador **15** *Lophocampa flavodorsata* sp. n., Holotype **16** *Lophocampa sullivani* sp. n., Holotype **17** *Lophocampa griseidorsata* sp. n., Holotype **18** *Lophocampa herbini* sp. n., Holotype.

**Figures 19–24.**
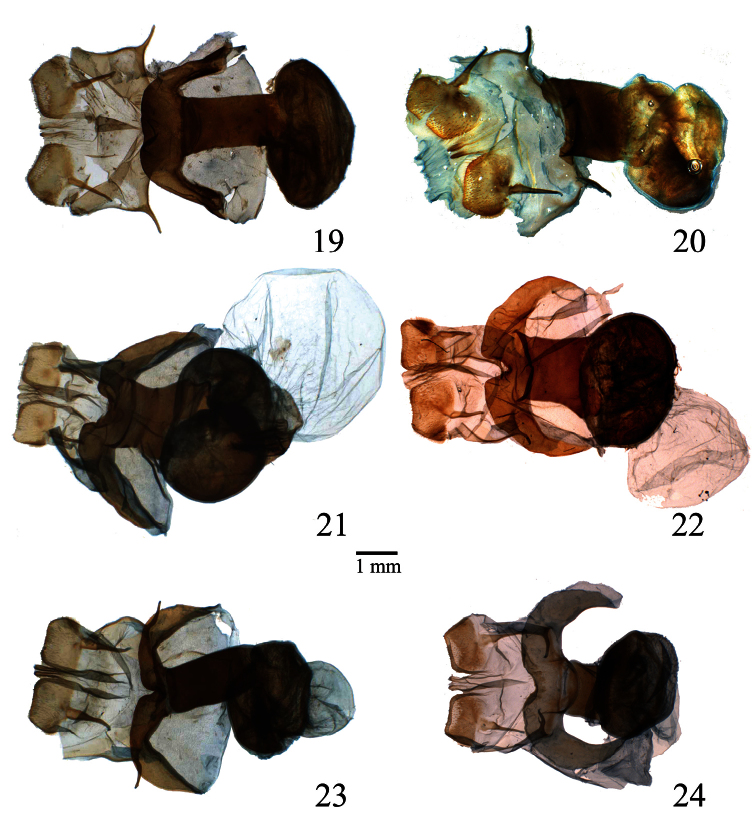
Female genitalia of *Lophocampa* species. **19** *Lophocampa atriceps* (Hampson, 1901), specimen from Ecuador **20** *Lophocampa hyalinipuncta* (Rothschild, 1909), specimen from Ecuador **21** *Lophocampa flavodorsata* sp. n., Allotype female **22** *Lophocampa sullivani* sp. n., Allotype female **23** *Lophocampa griseidorsata* sp. n., Allotype female **24** *Lophocampa herbini* sp. n., Allotype female.

Figs 1 , 7 , 13 and 19

Elysius atriceps Hampson, 1901: 113.

##### Type material.

*Elysius atriceps*: Male holotype [BMNH], examined. Type locality: Colombia. The holotype is labeled “*sychesia atriceps type ♂ Hmpsn*” (Hampson’s hand); “*Colombia 98-108*” and “*TYPE*” (typeset).

##### Redescription.

**Head** - Antenna bipectinate, brown with yellow base and brown cilia. Frons light yellowish white, separated at base of antenna by transverse black line. Vertex yellowish white with a prominent black central spot. Palpi erect, yellow with second joint black at sides and third black and very short. **Thorax -** Patagia yellowish white with square black spot. Tegulae exteriorly yellowish white, medially brown, bordered with black. Thorax yellow-white with posterior medial brown spot. Legs with femur dull white spotted with brown; dorsal face of femur vivid yellow. Tibia dull white with brown spots. Tarsal segments brown with small whitish spots. **Forewing -** Brown irrorated with light and dark brown. One yellow spot and two small blacks dots at base. A series of bands formed by whitish spots, arranged as follows: antemedial band comprised of three spots; medial band curved, comprised of seven dark spots; postmedial band sinuous, a series of different size spots, the three adjacent to costa largest. Three spots forming triangle between median and postmedian bands, outermost on costa. Subterminal band comprised of spots of different shapes: arrowhead-shaped spot nearest tornus, second spot larger, spots above veins CuA1 and M2 very small and flattened, three near apex large, last one on apex ovate and compressed basad. Terminal line a series of white dots along margin. Ventral markings as above but paler. **Hindwing -** White slightly tinged with yellow marks on apex and along costa, with yellow tint along the anal border. Markings on ventral surface more contrasting. **Abdomen -** Tergites pale orange, almost entirely covered with long yellow hairs, and with a lateral series of black spots. Last segment pale orange, without hair. Sternites white with yellow-centered brown patches. **Male genitalia -** Uncus lozenge-shaped, setose, apex truncate. Tegumen and vinculum very slender. Saccus tongue-shaped, folded ventrally. Valve symmetrical, enlarged at base, slightly exceeding apex of uncus. Valvula slender, oblong and slightly twisted internally. Cucullus short, pointed and folded toward uncus. Juxta slender, with two arms fused in middle forming a stem with a elongated sclerotized patch at apex. Transtilla with two arms folded and converging, arms not fused, showing a large disconnect in the structure. Penis rectilinear, narrow with elongated caecum penis. Vesica large with two large and one small diverticulum. Five patches of cornuti: one at base, one on small diverticulum, and one on each of two large diverticuli. **Female** - identical to male except as follows: wingspan slightly larger. Antennae with pectinations shorter than in male; two small black points laterally on the collar, the central pair always present. **Female genitalia -** Lamella antevaginalis subrectangular, lightly sclerotized. Apophyses posteriores straight, enlarged at base, 1.3 mm long; apophyses anteriores slightly curved, 0.75 mm long. Papillae anales rectangular, setose, with a pair of small pseudopapillae. Dorsal sac-like pheromone glands absent. Ductus bursae rectangular, striped. Corpus bursae ovate, wrinkled, with large signum. Insertion of ductus seminalis ventrad and close to juncture of ductus bursae with corpus bursae.

##### Biology and distribution.

Early stages unknown. Colombia (Valle del Cauca), Ecuador (Guayas) and Costa Rica (Alajuela, Guanacaste, Puntarenas, Cartago). Hampson associated with the holotype an unspecified number of specimens from Bolivia, R[io] Tanampaya, [La Paz]. A second male specimen in the BMNH labeled *atriceps* is a specimen of *Lophocampa griseidorsata* Vincent & Laguerre, sp. n.

##### Remarks.

The holotype in the BMNH has not been dissected. The type locality on the label states only “Colombia”, so it is not possible to compare specimens from the same locality. However, the tips of the valves of the holotype are visible, and were compared with those of superficially identical specimens which were dissected. Specimens with valves matching those of the holotype are from the western slopes of the Andes in Colombia and Ecuador and the mountains of Costa Rica. The description for the genitalia of *Lophocampa atriceps* provided here is based on a male originating from the western slope of Ecuador. A female from the same locality in Ecuador with similar habitus is described and illustrated, including the genitalia.

#### Lophocampa flavodorsata

Vincent & Laguerre sp. n.

urn:lsid:zoobank.org:act:DF055548-5A76-4621-8041-B98A35C750F0

http://species-id.net/wiki/Lophocampa_flavodorsata

Figs 3 , 9 , 16, 18 27

##### Type material.

**Holotype** - ♂, Ecuador, Napo, Route de Baeza à Tena, Km 7, 1.8 km after intersection on the right, 2097 m, 18-II-2006, 0.585˚S 77.877˚W, genitalia dissected by B. Vincent. n° BV 286 (fig. 9), Barcode ID ARCTA930-09, Sample ID BEVI0825, B. Vincent leg, [MNHN]. **Allotype** - ♀, same data as holotype, B. Vincent leg, [MNHN]. **Paratypes** - Venezuela: 1 ♂, Aragua, Geremba, 2050 m, 13-V-1994 P. Rouche; 1 ♂, Aragua, Road Colonia Tovar - Pto Maya, PK 4, 2100 m, 29-VII-1995, genitalia dissected by M. Laguerre n° ML 1151, M. Laguerre leg. ; 1 ♂, Aragua, Road Colonia Tovar - Pto Maya, PK 5, 2200 m, 30-VII-1995, M. Laguerre leg. ; 1 ♂, Aragua, Road Colonia Tovar - Pto Cruz, PK 4, 1800 m, 15-VIII-1995, M. Laguerre leg. all [MLC] ; 1 ♂, Lara, Parc National Yacambu, 1650 m, 17-XI-2001, genitalia dissected by B. Vincent. n° BV 229, B. Vincent leg. 1 ♂, Merida, Road la Azulita - Lagunillas, Km 15, 1700 m, 12-XI-2001, genitalia dissected by B. Vincent. n° BV 101, B. Vincent leg.; 2 ♂, Trujillo, Road Bocono - Biscucuy, Km 7, 1900 m, 10-XI-2001, genitalia dissected by B. Vincent. n° BV 227, B. Vincent leg. all [BVC] ; Ecuador: 1 ♂, Morona-Santiago, Road Gualaquiza - Limon, Km 23, 1610 m, 11-II-83, C. Lemaire & P.Thiaucourt leg., genitalia dissected by H. de Toulgoët. n°AS 38, [MNHN] ; 1 ♂, Sucumbios, Road Julio Andrade - La Bonita, PK 57, 2100 m, 3-VIII-1997, genitalia dissected by M. Laguerre. n° ML 1152 + 1 ♀ genitalia dissected by M. Laguerre. n° ML 1063, M. Laguerre leg. ; 1 ♀, Napo, Road Cosanga - Tena, PK 15, 1900 m, 5-VIII-1997, Barcode ID ARCTA838-07, Sample ID MILA0557, M. Laguerre leg. ; 1 ♂, Napo, Cordillera Huacamayos, 1850 m, XI-1992, genitalia dissected by M. Laguerre. n° ML 314, Barcode ID ARCTA839-07, Sample ID MILA0558, T. Porion leg. ; 2 ♂, Napo, Road Baeza - Tena, Km 17, 1.8 km after intersection on the right, 2097 m, 18-II-2006, S0.585, 77.877W, Barcode ID ARCTA330-07, Sample BEVI0048, Barcode ID ARCTA538-07, Sample ID BEVI0256 and Barcode ID ARCTA930-09, Sample ID BEVI0275, B. Vincent leg. ; 1 ♂, Napo, Route Cosanga - Tena PK 8, 2300 m, 19-VII-1990, Jean Haxaire & Daniel Herbin leg. Barcode ID ARCTA556-07, Sample BEVI0274, all [BVC] ; Peru: 1 ♂, Cuzco, Road Cuzco - Manu, PK 151, 1650 m, 15/18-XII-1979, T. Porion leg. genitalia dissected by B. Vincent. n° BV 251 ; 1 ♀, idem, genitalia dissected by B. Vincent. n° BV 28 ; 1 ♂, Road Lima - Pucallpa, 30 km after Tingo-Maria, 1000 m, 19 and 20-XI-1979, T. Porion leg, genitalia dissected by B. Vincent, n° BV 252, all in MNHN.

##### Etymology.

The specific epithet, *flavodorsata*, refers to the yellowish hairs of the abdominal tergites.

##### Diagnosis.

*Lophocampa flavodorsata* sp. n. can be distinguished from *Lophocampa atriceps* by the absence of black dots on the collar, the dark color of the head, collar and the base of the tegulae, as well as the different structure of both the male and female genitalia.

##### Description.

**Head** - Antenna bipectinate, brown with yellowish base and brownish cilia. Frons light yellow on inferior half, deep brown on superior half. Vertex light yellow with strong brown central spot. Palpi erect, deep brown with second segment light yellow and third very short. **Thorax -** Patagia light yellow with a square brown spot centered with brownish orange. Tegulae exteriorly brown, medially yellow-white, bordered with dark brown. Thorax light yellow with a median brown spot. Legs: Femur white spotted with brown, dorsal surface vivid yellow. Tibia white with brown spots. Tarsal segments brown with small white spots. **Forewing -** Brown irrorated with light and dark brown: one yellow spot with two small black dots at base. A series of bands formed by white spots arranged as follows: antemedial band broken; medial band slightly curved; postmedial band sinuous, originating at anal border and splitting between veins CuA2 and CuA1 into two branches reaching costa. After their separation the branches surround a strong brown reniform spot. Size of spots in this band relatively constant. Basal branch slightly contrasting due to clear background. Subterminal band made up of different-shaped spots: spot near tornus arrowhead shaped, 2nd spot larger than first, those above veins CuA1 and M2 very small and flattened; three spots near apex larger; last one at apex ovate and compressed basally. Terminal line of white dots on margin. Ventrally as above but paler. **Hindwing -** Yellow-white slightly tinged with yellow-brown marks on apex and along the costa, with yellow tinge along anal border. Ventrally, marks more contrasting, deep brown centered with yellow-brown. **Abdomen -** Tergites pale orange and almost entirely covered with long yellow hair, with a lateral series of very faint brown spots. Last segment pale orange, without hair. Sternites white with brown patches centered with yellow. **Male genitalia -** Uncus rectangular, setose. Tegumen and vinculum slender. Saccus tongue-shaped, folded ventrally. Valves symmetrical, slender and long with apex slightly angled, extending beyond apex of the uncus. Valves slender, very elongate, terminating in a small tooth. Cucullus slender, elongated, folded toward uncus. Juxta very narrow, with two arms fused apically into a small V-shaped sclerotized patch. Transtilla slender, tongue-shaped, interrupted in the middle. Aedoeagus. Penis rectilinear; caecum penis present. Vesica wide with four diverticuli, with four patches of cornuti, two with small spines and two with larger spines. **Female** - identical to male except as follows: Antennae with pectinations shorter than male. **Female genitalia** - Lamella antevaginalis slightly sclerotized, semicircular with a large medial U-shaped notch on the posterior margin. Apophyses posteriores straight, 0.9 mm long, apophyses anteriores slender, 0.6 mm long. Papillae anales rectangular, setose, with a pair of small pseudopapillae. Dorsal saccular pheromone glands absent. Ductus bursae asymmetrical with an extension on the right (ventral view). Corpus bursae reduced and ovate, bearing a large field of signa. Appendix bursae as large as corpus bursae. Ductus seminalis ventrally inserted at apex of corpus bursae and immediately transformed into seminal vesicle.

##### Biology and distribution.

Early stages unknown. Venezuela (Aragua, Lara, Merida), Ecuador (Napo, Morona-Santiago, Sucumbios), Peru (Cuzco, Huanuco).

#### Lophocampa griseidorsata

Vincent & Laguerre sp. n.

urn:lsid:zoobank.org:act:A4BEFCB0-2305-4C8F-9881-7FD02C377880

http://species-id.net/wiki/Lophocampa_griseidorsata

Figs 5 , 11, 17 , 23 29

##### Type material.

**Holotype** - ♂, Bolivia, Sud Yungas, Rte Caranavi - Palos Blancos, K 41, 1400 m 16-III-84, G. Lachaume & T. Porion leg., genitalia dissected by B. Vincent n° BV 257, Barcode ID ARCTA931-09, Sample ID BEVI0826 [MNHN]. **Allotype** - ♀, Bolivia, Sud Yungas, Rte Caranavi - Palos Blancos, K 41, 1400 m 16-III-84, G. Lachaume & T. Porion leg., genitalia dissected by B. Vincent n° BV 280 [MNHN]. **Paratypes** - 1 ♂, Bolivia: Cochabamba, Road Cochabamba - Villa Tunari, PK 103, 2100 m, 28-I-1997, genitalia dissected by M. Laguerre. n° ML 1180, M. Laguerre leg. ; 2 ♀, Cochabamba, Road Cochabamba - Villa Tunari, PK 111, 1500 m, 29-I-1997, genitalia dissected by M. Laguerre. n° ML 1064, M. Laguerre leg. all [MLC] ; 1 ♂, Coroico, 1700 m, XII-1981, G. Jeannot & G. Lachaume leg. genitalia dissected by B. Vincent n° BV 254, [MNHN] ; Peru: 1 ♂, Road Olmos - Tarapoto, 1700 m, 10/12 -I-80, T. Porion leg., with one genitalia dissected by B. Vincent n° BV 256, [MNHN] ; 2 ♂ and 1 ♀, Cuzco, Road Cuzco-Manu PK 151, 1650 m, 15/18-XII-79, T. Porion leg., genitalia dissected by B. Vincent n° BV 255, [MNHN]; 1 ♀, Pasco, Route d’Oxopampa - Pozuzo, km 47, 1500 m, 30-IX-2003, B. Vincent leg., Barcode ID ARCTA929-09, Sample ID BEVI0824 all [BVC] ; Ecuador: 3 ♂, Sucumbios, Road Julio Andrade - La Bonita, PK 57, 2100 m, 3-VIII-1997, genitalia dissected by M. Laguerre. n° ML 317 & 1156, M. Laguerre leg. [MLC]; 1 ♂, Napo, Road Baeza - Tena, Km 17, 1.8 km after intersection on the right, 2097 m, 18-II-2006, S0.585, 77.877W, B. Vincent leg., Barcode ID ARCTA538-07, Sample ID BEVI0256 ; 1 ♂, Morona-Santiago, Road Limon - Gualaceo, Km 28.6, 2114 m, 22-II-2006, S3.022, 78.586W, Barcode ID ARCTA539-07, Sample ID BEVI0257, B. Vincent leg. [BVC]; 1 ♂, Road Gualaceo - Mendez, Km 49, 1700 m, 23-I-1979, T. Porion leg, genitalia dissected by H. de Toulgoët. n°AS 55, [MNHN].

##### Etymology.

The specific epithet *griseidorsata* refers to the gray color of the abdominal tergites.

##### Diagnosis.

*Lophocampa griseidorsata* sp. n. is best distinguished from *Lophocampa flavodorsata* by the difference in the coloration of the abdominal scales, the larger whitish spots on forewings and by the structure of male and female genitalia, as given in the key. This species can also be distinguished from *Lophocampa atriceps* with the characters above along with the absence of black spots on the collar.

##### Description.

**Head** - Antenna bipectinate, brown with basal third vivid yellow and with brownish cilia. Frons whitish with black T-shaped mark, vertex whitish anteriorly and black centered with brown posteriorly. Palpi erect, deep brown with 2nd segment whitish and 3rd segment very short. **Thorax -** Patagiae whitish with square brown spot centered with brownish orange. Tegulae exteriorly whitish, medially brown, bordered with dark brown. Thorax whitish with median brown line. Legs. Femur whitish, spotted with brown and with dorsum vivid yellow. Tibia whitish, spotted with brown. Tarsal segments brown with small whitish spots. **Forewing -** Brown irrorated with pale and deep brown. At base, one whitish spot with a yellow and two small blacks dots. Series of bands formed by whitish spots and organized as follows: broken antemedial band; slightly curved medial band, the size of the spots decreasing from anal border to the costa; sinuous postmedial band starts on the anal border and splits between veins CuA2 and CuA1 into two branches that reach the costa. Size of spots constant on basal portion and basal branch. Apical branch constituted of larger spots. After their separation branches surround a strong brown reniform spot. Subterminal band constituted of different-shaped spots: spot near tornus forms a <-mark, 2nd spot larger, spots above veins CuA1 and M2 very small and flattened; three spots near apex large; last one at apex is large and ovate. A terminal line of white dots on the margin. Ventrally, the ornamentation is the same but paler. **Hindwing -** Whitish slightly tinged with grey on apex and along the costa. Ventrally, the marks are more contrasted, deep brown centered with yellowish brown. **Abdomen -** Tergites pale orange and almost entirely covered with long grayish brown hairs, with lateral series of black spots. Last segment pale orange without hairs. Sternites brown. **Male genitalia -** Uncus spatulated and setose. Tegumen and vinculum very slender. Saccus tongue shaped and folded ventrally. Valvae symmetrical, wide, reaching uncus apex. Valvae with prominent costa and acute, bifid apex, one tip short and rounded, the other very long and acute. Juxta with two curved arms fused apically to give a crescent-like sclerotized patch. Transtilla with two arms moving away at apex and which are not fused. Aedoeagus. Penis narrow and rectilinear. Caecum penis present. Vesica with a small and a large area of cornuti distally. **Female** - (Description based on allotype): identical to male excepted the following differences: Antennae with pectinations shorter than male. Wingspan slightly larger. **Female genitalia** - Lamella antevaginalis slightly sclerotized, rectangular with a medial notch on the posterior margin. Apophyses posteriores straight, enlarged at base, 1.2 mm long. Apophyses anteriores slightly curved, slender and 0.7 mm long. Papillae anales rectangular and setose, with a pair of large pseudopapillae. Dorsal saccular pheromone glands absent. Ductus bursae rectangular and striped. Corpus bursae reduced, oval, very wrinkled. Appendix bursae slightly smaller than corpus bursae, without signa. Apical insertion of the ductus seminalis.

##### Biology and distribution.

Rab Green et al. (2011) described and figured the larva of this new species under the name *Lophocampa atriceps*. The comparison with the male genitalia of the studied specimen confirms it belongs to *Lophocampa griseidorsata* sp. n. (Rab-Green pers. comm.). According to Rab Green et al. (2011), the larva feeds on several species of Urticaceae, but also on Poaceae, Fabaceae, Ericaceae, Melanostomaceae, and Rubiaceae. Distribution: Bolivia (Cochabamba, La Paz), Ecuador (Napo, Morona-Santiago, Sucumbios, Azuay), Peru (San Martin, Cuzco, Pasco).

#### Lophocampa herbini

Vincent & Laguerre sp. n.

urn:lsid:zoobank.org:act:17E08972-5886-4A15-8F70-A4426AE06342

http://species-id.net/wiki/Lophocampa_herbini

Figs 6 , 12 , 18 , 24 30

##### Type material.

**Holotype** - ♂, Bolivia, Santa Cruz, Road Samaipata to Santa Cruz, Km 6, 2037 m, 14-XI-2007, 18.118˚S 63.801˚W, Barcode ID ARCTA926-09, Sample ID BEVI0821, B Vincent leg., genitalia dissected by B Vincent n° BV 351 [MNHN]. **Allotype** - ♀, Bolivia, Cochabamba, Road Cochabamba - Villa Tunari, PK 85, 2400 m, track to Inca Chaca, PK 4, 14-II-1997, M. Laguerre leg. genitalia dissected by M. Laguerre n° ML 1181 [MNHN] ; **Paratypes** - Bolivia: 3 ♂ + 1 ♀, Cochabamba, Road Cochabamba - Villa Tunari, PK 87, 2000 m, 15-II-1997, genitalia dissected by M. Laguerre n° ML 1155, ML 310, Barcode ID ARCTA179-07, Sample ID MILA0179, M. Laguerre leg. 1 ♂, La Paz, Road Chulumani - Inquisivi, PK 100, 2170 m, 21-X-2000, M. Laguerre leg., genitalia dissected M. Laguerre n° ML 1049, Barcode ID ARCTA073-07, Sample ID MILA0073, all [MLC] ; 3 ♂, Santa Cruz, Road Comarapa to Pojo, Km 26.8, 2548 m, 13-XI-2007, S17.824, 64.686W, Barcode ID ARCTA601-08, ARCTA927-09, ARCTA928-09, Sample ID BEVI0511, BEVI0822 and BEVI0823, B Vincent leg. [BVC] ; Peru: 6 ♂, Cuzco, Road Cuzco to Quillabamba, PK 177, 1.3 km after Alfamayo, 2395 m, 20-II-2009, S13.061, 72.413W, B. Vincent leg., 3 specimens in [BVC], 3 specimens will be deposited in [MUSM].

##### Etymology.

*Lophocampa herbini* is named in honor of Daniel Herbin, specialist of Saturniidae and friend of the authors.

##### Diagnosis.

*Lophocampa herbini* sp. n. is best distinguished from *Lophocampa atriceps* and *Lophocampa flavodorsata* sp. n. by the difference in the coloration of the abdominal pilosity, the larger whitish spots on forewings and by the conformation of male and female genitalia. This species can also be distinguished from *Lophocampa griseidorsata* with the same characters along with the absence of black spots on the collar. It is also the only species with a thin black line crossing the white ovate apical spot on the forewing.

##### Description.

**Head** - Antenna bipectinate, brown with basal third vivid yellow and with brownish cilia. Frons whitish with a black T-shaped mark, vertex whitish anteriorly and black centered with brown posteriorly. Palpi erect, deep brown 2^nd^ segment whitish, 3rd segment very short. **Thorax -** Patagia whitish with a square brown spot centered with brownish orange. Pterygodes exteriorly whitish, medially brown, bordered with dark brown. Thorax whitish with a median brown line. Femur whitish, spotted with brown, dorsum vivid yellow. Tibia whitish, spotted with brown. Tarsal segments brown with small whitish spots. **Forewing -** Brown irrorated with pale or deep brown. At base, one whitish spot with one yellow and two small blacks dots. Presence of a series of bands formed by whitish spots and organized as follows: a broken antemedial band. A slightly curved medial band. The size of the spots decreasing from anal border to the costa. A sinuous postmedial band starts on the anal border and splits between veins CuA2 and CuA1 into 2 branches that reach the costa. The size of the spots is constant on the basal portion and the basal branch. The apical branch is constituted by larger spots. After their separation the branchs surround a strong brown reniform spot. Ssubterminal band constituted of different-shaped spots: spot near tornus forming a <-mark, 2nd spot larger, spots above veins CuA1 and M2 very small and flattened; three spots near apex large, last one on apex ovate. Terminal line of white dots on margin. Ventral pattern similar to dorsal, but paler.

**Hindwing -** Whitish, slightly tinged with grey on apex and along the costa. Ventral pattern more contrasting, deep brown centered with yellowish brown. **Abdomen -** Tergites pale orange, almost entirely covered with long grayish brown hairs, with lateral series of black spots. Last segment pale orange without hairs. Sternites brown. **Male genitalia -** Uncus setose, lozenge shaped, apex truncated and blunt. Tegumen and vinculum very slender. Saccus virtually absent. Valvae symmetrical, wide, reaching uncus apex, without prominent costa. Apex of valvae bifid, the two tips symmetrical, short and acute. Juxta with two curved arms fused apically to give a crescent-like sclerotized patch. Transtilla with two semi-rounded arms which are not fused. Aedoeagus. Penis moderately narrow and rectilinear. Caecum penis present. Vesica wide with four diverticuli and four spine patches: two with small spines and two with strong spines. **Female** - (description based on allotype): identical to male excepted the following differences:

Antennae with pectinations shorter than male. Wingspan slightly larger. **Female** **genitalia** - lamella antevaginalis slightly sclerotized, semicircular with a large medial median U-shaped notch on the posterior margin. Apophyses posteriores straight, enlarged at base, 0.9 mm long. Apophyses anteriores narrower, slightly curved and 0.6 mm long. The left apophysis (ventral view) displays a fork at its apical extremity. Papillae anales rectangular and setose with a pair of small pseudopapillae. Dorsal saccular pheromone glands absent. Ductus bursae short, square and striate. Corpus bursae ovate, bearing a large signa. Ductus seminalis ventrally inserted at the extremity of a wide projection of corpus bursae and immediately transformed into a seminal vesicle.

##### Biology and distribution.

Early stages unknown. Distribution: Bolivia (Santa Cruz, Cochabamba), Peru (Cuzco).

#### Lophocampa sullivani

Vincent & Laguerre sp. n.

urn:lsid:zoobank.org:act:428502AC-C12A-480D-B147-5C759716E1BA

http://species-id.net/wiki/Lophocampa_sullivani

Figs 4 , 10 , 16 , 22 28

##### Type material.

**Holotyp**e - ♂, Ecuador, Pichincha, Road Chiriboga- Santo Domingo de los Colorados, Km 11, Réserve Rio Gualajito, 1969 m, 01-III-2006, 0.249°S 78.817°W, Barcode ID ARCTA933-09, Sample ID BEVI0828, B. Vincent leg., genitalia dissected by B. Vincent n° BV 289, [MNHN]. **Allotype** - ♀, Ecuador, Pinchicha, Rte Nono – Nanegalito, Km 25, 1840 m, 10-I-1983, C. Lemaire and N. Venedictoff leg., genitalia dissected by B. Vincent n° BV 282, Barcode ID ARCTA934-09, Sample ID BEVI0829, [MNHN]. **Paratypes** - Ecuador: 1 ♂, Pichincha, Road Nono -Nanegalito, pK 25, 1850 m, 30-VII-1997, Barcode ID ARCTA840-07, Sample ID MILA0559, M. Laguerre leg., genitalia dissected by M. Laguerre n° ML 316 ; 1 ♂, same data, genitalia dissected by M. Laguerre n° ML 1224, all [MLC] ; Colombia: 2 ♂, Valle del Cauca, Finca Monte Bello, 5 km South of Bitaco, 30-I-1988, 2000 m, Bo Sullivan leg. [BSC].

##### Etymology.

*Lophocampa sullivani* is named in honor of J. Bolling Sullivan who collected this species in 1988.

##### Diagnosis.

*Lophocampa sullivani* sp. n. can be best distinguished from *Lophocampa atriceps* by the absence of black dots on the collar and by the structure of the genitalia. It can be distinguished from *Lophocampa griseidorsata* sp. n. and *Lophocampa herbini* sp. n. by the difference in color of the abdominal scales. *Lophocampa sullivani* sp. n. is very close in appearance to *Lophocampa flavodorsata* sp. n. but can be separated by characters of the male genitalia, including the very prominent cucullus on the valve, the more robust genitalia and the presence of only three spine fields on the vesica. The female genitalia appear to be identical to those of *Lophocampa flavodorsata*.

##### Description.

**Head** - pale yellowish, vertex brown with horizontal yellow bar, palpi dark fuscous with 2nd segment yellowish orange; antenna yellowish brown with brownish ciliae. **Thorax -** Patagia yellowish brown with a square brown spot centered with yellow, pterygodes striated with yellowish brown and chestnut brown, thorax yellowish with admixture of brown hairs. Legs yellowish, spotted with chestnut brown, fore coxae orange. **Forewing -** Yellowish irrorated with chestnut brown and a series of creamy-white spots with diffuse brown edges. At base, one orange spot with two small black dots. An antemedial, very oblique series of three creamy-white spots; a medial series angled on median vein, then very oblique, the two spots towards costa small; a spot at end of cell; an oblique postmedial series of six spots starting from anal border extending to end of cell followed by two series of three large spots, one basad and one distad, the last one just on the apex has the bottom constricted; a subterminal series of four spots, one near tornus forming a <-mark, the 2nd large, those above veins 3 and 4 smaller; a terminal line of white dots on the margin. **Hindwing -** Yellowish white slightly tinged with fuscous on apex and along the costa. **Abdomen** - entirely yellowish, with lateral series of black spots and ventral surface whitish. **Male genitalia -** Genitalia very robust. Uncus rectangular and setose, with slightly rounded apex. Tegumen and vinculum slender. Saccus tongue shaped and folded ventrally. Valvae symmetrical, wide and relatively short with apex notched into two points: one blunt and one acute. Cucullus prominent, slender and elongate, folded inward. Juxta very narrow, with two arms fused apically in a small V-shaped sclerotized patch. Transtilla slender, shaped like a tongue interrupted in the middle. Penis rectilinear. Caecum penis present. Vesica wide with four lobes. Presence of three areas of cornuti, two densely covered with small spines and one sparsely covered with strong spines. **Female** – (description based on allotype): identical to male except for antennae with pectinations shorter than male and wingspan slightly larger. **Female genitalia** - identical to those of *Lophocampa flavodorsata*.

##### Biology and distribution.

Early stages unknown. Distribution: Ecuador (Pichincha), Colombia (Valle del Cauca).

#### Lophocampa andensis

(Schaus, 1896)

http://species-id.net/wiki/Lophocampa_andensis

Halisidota andensis Schaus, 1896: 138

##### Type material.

Lectotype male n° 11079 designated by Watson 1973: 7 [USNM] examined. Type locality: Colombia. The genitalia was dissected by A. Watson (slide N° AW 626 in USNM). The lectotype specimen and genitalia are figured in Watson (1973).

##### Diagnosis.

*Lophocampa andensis* is the smallest species of the group, with the length of the male forewing less than 20 mm. It is also distinguished from other species by the rounded apex of the forewings and by the spots between M2 and CuA1 on the forewing subterminal band, which are at least as large as the others.

###### Biology and distribution.

Early stages unknown. Distribution: Only known from Colombia.

#### Lophocampa hyalinipuncta

(Rothschild, 1909)

http://species-id.net/wiki/Lophocampa_hyalinipuncta

Figs 2 , 8 , 14 20

Halisidota hyalinipuncta Rothschild, 1909: 217.

##### Type material.

Described from four male syntypes [BMNH]. Type locality: [Peru], [Puno], Agualani, Carabaya. One specimen of this syntype series is labelled ‘TYPE’. The same specimen is labelled “Halisidota hyalinipuncta Rothschild Type” on a pink label in Rothschild’s hand.

##### Diagnosis.

*Lophocampa hyalinipuncta* can be distinguished from *Lophocampa atriceps*, *Lophocampa flavodorsata* sp. n. and *Lophocampa sullivanni* sp. n. by the different pattern of scales on the **Abdomen -** The spots of the subterminal band of the forewing are also significantly smaller than the spots on the postmedial band, a character that separates *hyalinipuncta* from the other species of the group.

##### Biology and distribution.

Early stages unknown. Distribution: Ecuador (Morona-Santiago, Carchi); Peru (Huanuco, Puno, Amazonas); Bolivia (La Paz, Cochabamba, Chuquisaca).

## Discussion

**Figure 25.**
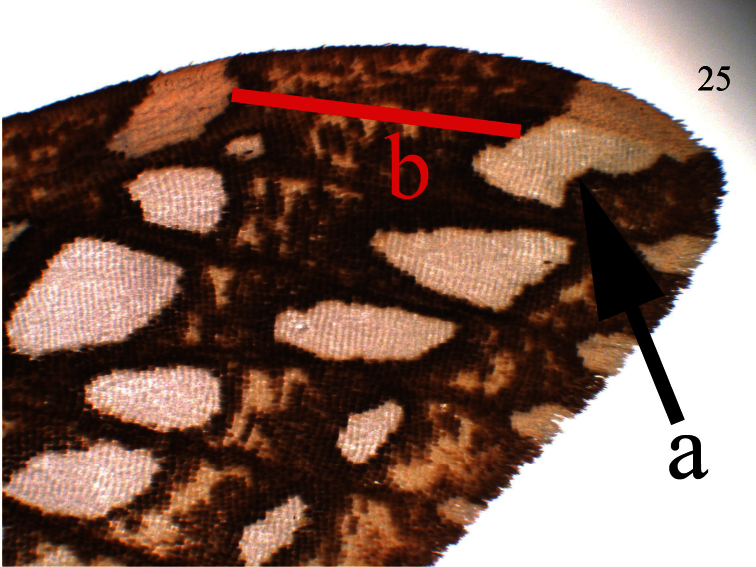
*Lophocampa flavodorsata* sp. n. forewing apex.

**Distribution patterns.** Each of the five species is restricted to only one slope of the Andean Cordillera (Table 1; Fig. 25); *Lophocampa atriceps* and *Lophocampa sullivani* are restricted to the western slope; *Lophocampa flavodorsata*, *Lophocampa griseidorsata*, *Lophocampa herbini* occur only on the eastern slope. *Lophocampa atriceps* and *Lophocampa sullivani* occur at different elevations, with *Lophocampa sullivani* at elevations of 700–1450 m, and *Lophocampa atriceps* from 1840 - 2000 m, which will help separate the two taxa. The three taxa occurring on the eastern slope of the Andes overlap in range, and *Lophocampa flavodorsata* and *Lophocampa griseidorsata* have been collected several times at the same locality. The *atriceps* group occupies a very large area of the Andean Cordillera and extends north to the central mountain range in Costa-Rica at medium to high altitudes.

**Figure 26.**
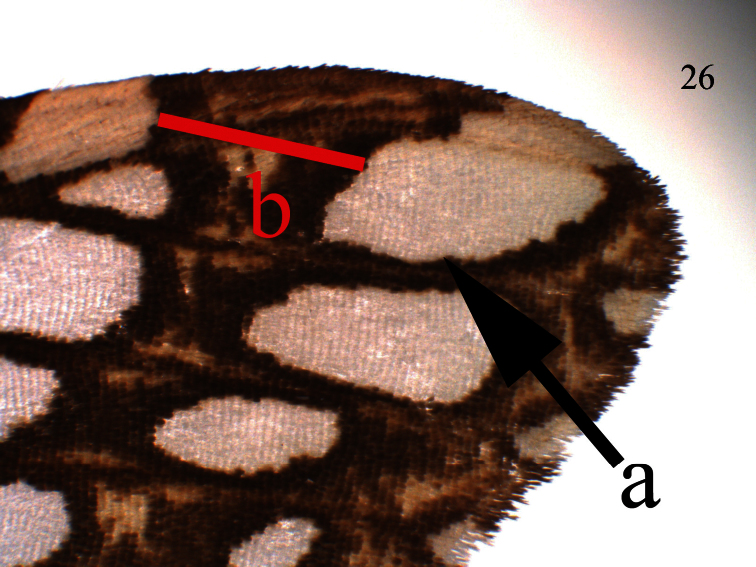
*Lophocampa griseidorsata* sp. n. forewing apex.

**Figures 27–30.**
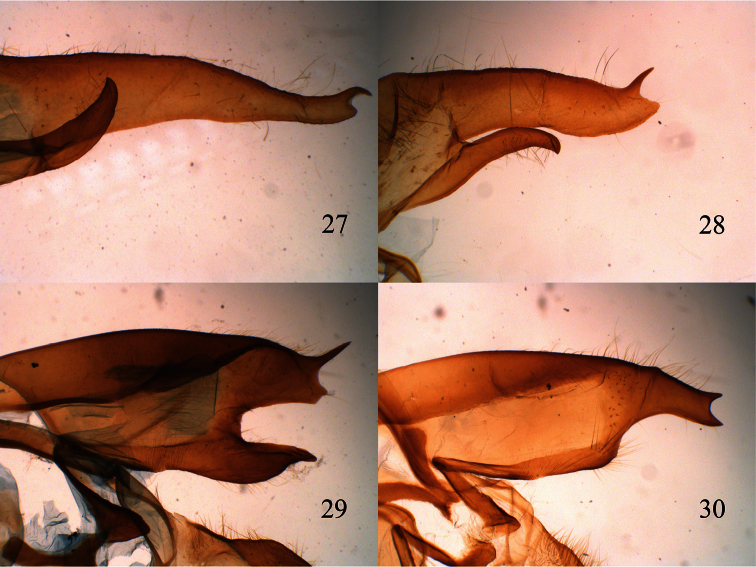
lateral view of male valve. **27** *Lophocampa flavodorsata* sp. n. **28** *Lophocampa sullivani* sp. n. **29** *Lophocampa griseidorsata* sp. n. **30** *Lophocampa herbini* sp. n.

**Figure 31.**
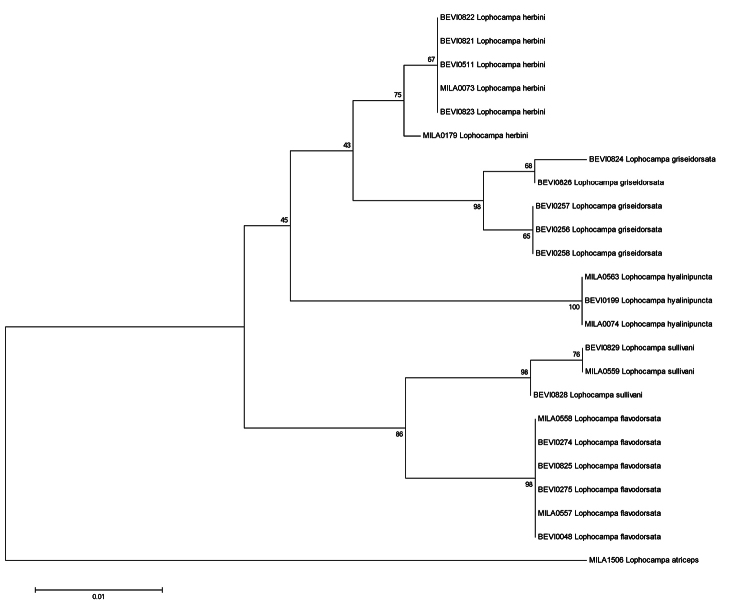
Neighbor-Joining Tree for the 24 specimens of the *Lophocampa atriceps* group. Boot-strap values (in %, 1000 replicates) are given on each branch (obtained with MEGA5, see Tamura et al. 2007).

**Figure 32.**
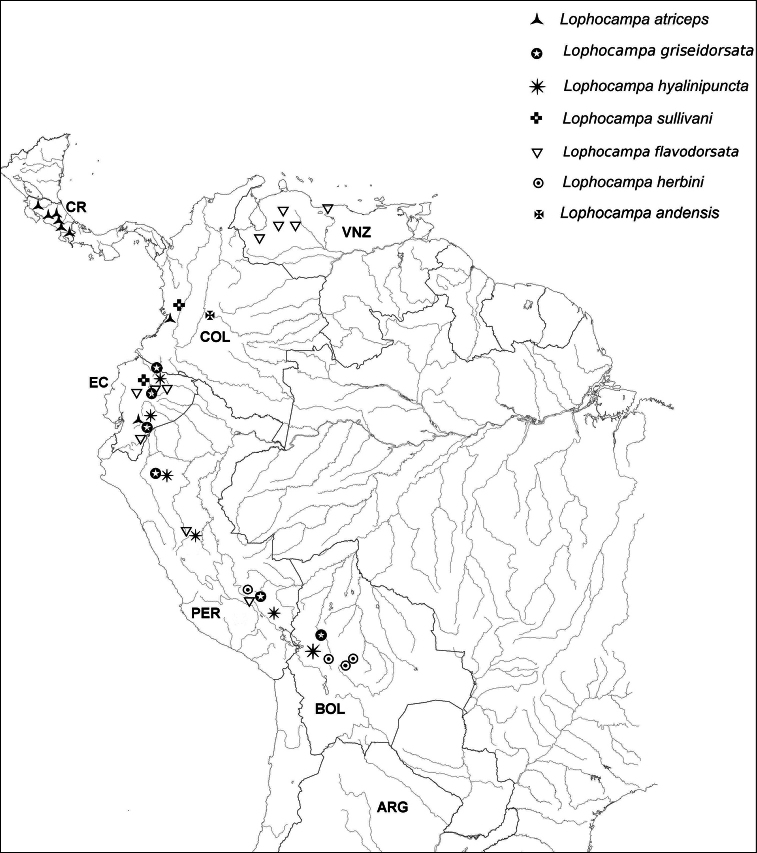
Distribution of examined specimens of the *Lophocampa atriceps* group.

The Andean Cordillera appears to have created a barrier between the species studied here. *Lophocampa atriceps* is distributed from Central America in the north to Ecuador in the south but is restricted to the western slope in South America. A similar pattern has been observed for several plant species (Schnell 1987). In northern Peru, the presence of a very dry region on the Pacific slope prevents a more southerly range extension.

**Molecular data.** Twenty-four voucher specimens provided barcode sequences (Table 2). Calculations of genetic distances between species of the *Lophocampa atriceps* group (Table 3) support the distinctness of the five species. Intraspecific divergence ranged from 0 – 0.99 % with a mean divergence of 0.21 %. Interspecific divergence ranged from 2.02 – 5.46 % with a mean divergence of 3.61 %.

**Table 2.** Samples used for the molecular analyses "COI" refers to the barocde sequence length, holotypes in bold.

**Species**	**Country, Province**	**Collection**	**COI**	**SampleID**	**GenBank**
***Lophocampa flavodorsata* sp. n.**	**Ecuador, Napo**	**MNHN**	**658**	**BEVI0825**	**JX887762**
*Lophocampa flavodorsata* sp. n.	Ecuador, Napo	BVC	658	BEVI0048	JX887766
*Lophocampa flavodorsata* sp. n.	Ecuador, Napo	BVC	502	BEVI0274	JX887765
*Lophocampa flavodorsata* sp. n.	Ecuador, Napo	BVC	657	BEVI0275	JX887764
*Lophocampa flavodorsata* sp. n.	Ecuador, Napo	MLC	267	MILA0557	JX887763
*Lophocampa flavodorsata* sp. n.	Ecuador, Napo	MLC	267	MILA0558	JX887761
***Lophocampa griseidorsata* sp. n.**	**Bolivia, La Paz**	**MNHN**	**307**	**BEVI0826**	**JX887769**
*Lophocampa griseidorsata* sp. n.	Ecuador, Napo	BVC	658	BEVI0256	JX887767
*Lophocampa griseidorsata* sp. n.	Ecuador, Morona-Santiago	BVC	658	BEVI0257	JX887770
*Lophocampa griseidorsata* sp. n.	Peru, Pasco	BVC	658	BEVI0824	JX887768
*Lophocampa griseidorsata* sp. n.	Ecuador, Napo	BVC	658	BEVI0258	JX887771
***Lophocampa herbini* sp. n.**	**Bolivia, Santa Cruz**	**MNHN**	**658**	**BEVI0821**	**JX887773**
*Lophocampa herbini* sp. n.	Bolivia, Santa Cruz	BVC	656	BEVI0511	JX887774
*Lophocampa herbini* sp. n.	Bolivia, Santa Cruz	BVC	658	BEVI0822	JX887772
*Lophocampa herbini* sp. n.	Bolivia, Santa Cruz	BVC	658	BEVI0823	JX887777
*Lophocampa herbini* sp. n.	Bolivia, La Paz	MLC	619	MILA0073	JX887775
*Lophocampa herbini* sp. n.	Bolivia, Cochabamba	MLC	269	MILA0179	JX887776
***Lophocampa sullivani* sp. n.**	**Ecuador, Pichincha**	**MNHN**	**658**	**BEVI0828**	**JX887782**
*Lophocampa sullivani* sp. n.	Ecuador, Pichincha	MNHN	307	BEVI0829	JX887781
*Lophocampa sullivani* sp. n.	Ecuador, Pichincha	MLC	267	MILA0559	JX887783
*Lophocampa hyalinipuncta*	Bolivia, Chuquisaca	BVC	578	BEVI0199	JX887778
*Lophocampa hyalinipuncta*	Bolivia, La Paz	MLC	619	MILA0074	JX887780
*Lophocampa hyalinipuncta*	Bolivia, La Paz	MLC	573	MILA0563	JX887779
*Lophocampa atriceps*	Costa Rica, Cartago	MLC	658	MILA1506	JX887760

**Table 3.** Mean Kimura-2-parameter distances for DNA barcode sequences calculated within and between each of the taxa included in the dataset.

	*Lophocampa flavodorsata* sp. n.	*Lophocampa griseidorsata* sp. n.	*Lophocampa herbini* sp. n.	*Lophocampa sullivani* sp. n.	*Lophocampa hyalinipuncta*	*Lophocampa atriceps*
*Lophocampa flavodorsata* sp. n.	0 %					
*Lophocampa griseidorsata* sp. n.	4,98%	0,99%				
*Lophocampa herbini* sp. n.	3.58%	2,02%	0.39%			
*Lophocampa sullivani* sp. n.	2.27%	4.58%	4.05%	0.39%		
*Lophocampa hyalinipuncta*	4.91%	4.14%	3.56%	5.19%	0 %	
*Lophocampa atriceps*	8,41%	8,55%	8,05%	9.64%	9,33%	-

The four new species described herein were all originally identified as *Lophocampa atriceps*. Differences in barcode sequences of *Lophocampa atriceps* and the four new species described herein range from 8.05 – 9.64 %. These values are significant and confirm the significance of the morphological differences observed and the discrimination of the taxa. The greatest difference (9.64 %) is observed between *Lophocampa atriceps* and *Lophocampa sullivani*, the unique sympatric species. The geographic isolation that occurred with the species on the eastern slopes of the Andes did not result in the highest divergence values.

The lowest interspecific divergence was observed between *Lophocampa griseidorsata* and *Lophocampa herbini* with 2.02 %. Morphologically both species are in fact the closest with similar male genitalia structure. They do not appear to be sympatric because their altitudinal difference is in the range of 400 m. Similarly low interspecific divergence is observed also between *Lophocampa flavodorsata* and *L sullivani*, with 2.27 %. Again the two species are morphologically similar and show little difference in male genitalia structure, but they occur on different slopes of the Andes. *Lophocampa hyalinipuncta* differs from the new taxa by 3.56 - 5.19 %. These values are significantly lower than for *Lophocampa atriceps* (9.33 %) even if the new species have hitherto been confused with *Lophocampa atriceps*. Specimens of *Lophocampa andensis* were not available for DNA sequencing.

The barcode data shows that *Lophocampa flavodorsata* is most closely related to *Lophocampa sullivani*, with each restricted to only one slope of the Andes, but with a broader geographic distribution for *Lophocampa flavodorsata*. On the other hand, the pair *griseidorsata* + *herbini* is closest to *hyalinipuncta* and well-differentiated from *flavodorsata* + *sullivani*. Here also one species (*griseidorsata*) has a broad distribution whereas the second (*herbini*) appears to have a very restricted range in Bolivia. *Lophocampa flavodorsata* and *Lophocampa griseidorsata* which share a broad range on the Atlantic slope of the Andes display the largest difference in the COI gene sequence (4.9–5.3 %) for the whole group along with the pair *sullivani* + *hyalinipuncta* (which seems normal for such widely differentiated species).

## Supplementary Material

XML Treatment for Lophocampa atriceps

XML Treatment for Lophocampa flavodorsata

XML Treatment for Lophocampa griseidorsata

XML Treatment for Lophocampa herbini

XML Treatment for Lophocampa sullivani

XML Treatment for Lophocampa andensis

XML Treatment for Lophocampa hyalinipuncta
